# Obesity: an heavyweight player in breast cancer’s chemoresistance

**DOI:** 10.18632/oncotarget.26905

**Published:** 2019-05-14

**Authors:** Charlotte Vaysse, Catherine Muller, Frédérique Fallone

**Keywords:** chemoresistance, breast cancer, obesity, adipocytes

While obesity is associated with postmenopausal breast cancer (BC) risk and is a negative prognostic factor influencing BC recurrence and survival [1], the underlying mechanisms of this effect are not well understood. One explanation is provided by an increased number of studies identifying obesity as a factor of resistance to anticancer therapy. For example, clinical data obtained from the Ewertz’s study show that the effects of adjuvant therapy (both chemotherapy and endocrine therapy) seem to be lost more rapidly in patients with BC and obesity [2]. Another explanation is the underdosing of therapy regimens in obese patients, due to the fear of toxicity, contributing to the decrease in treatment efficacy. A recent review by the American Society of Clinical Oncology [3] aims at circumventing this practice and recommend to use full weight-based chemotherapy doses, especially since there is no evidence of increased hematologic or non-hematologic toxicity with such dosing. In addition, obesity is also incriminated in modifying the pharmacokinetic of chemotherapy drugs thereby contributing to resistance as demonstrated in animal models [4]. Our hypothesis is that independently of these parameters, obesity could impact cancer resistance to therapies through inducing biological modifications of the adipose tissue (AT). We will detail the available results supporting this, with a focus on chemotherapy.

AT is a major component of the breast tumor microenvironment and numerous studies indicate that the mammary AT (MAT) adjacent to tumors supports BC development and progression [5]. In addition to their ability to store lipids, adipocytes are highly secretory cells, releasing a large panel of molecules named adipokines, including common growth factors, hormones, cytokines, chemokines, and more specific factors such as leptin and adiponectin [6]. Others and ourselves have shown that tumor-surrounding adipocytes stimulate cancer aggressiveness by secreting pro-inflammatory cytokines, extracellular matrix (ECM) and matrix metalloproteases and by modulating the cancer cell metabolism, processes likely to be amplified in obese patients as we reviewed [7]. Obese AT, related both to an increased adipose quantity and an alteration of adipose quality, has been characterized as being in a chronic inflammation state, with remodeling of local cellular composition and dysregulation of secreted adipokines [6]. As far as therapy resistance concerns, a growing number of studies indicate that adipocytes might protect tumor cells exposed to drugs, although this aspect is less documented than their effect on tumor aggressiveness. As we recently reviewed, adipocytes protect cancer cells by secreting adipokines, metabolites or exosomes, the main mechanisms involved being the modulation of cell death pathways [5]. Surprisingly, little is known about the mechanism involved in BC. Independently of obesity, adipocytes have been demonstrated to increase the pro-survival pathways of BC exposed to chemotherapy. For example, the adipokine resistin was shown to protect human BC cells from apoptosis induced by doxorubicin (DOX), a DNA-intercalating agent, through autophagy induction [8]. This effect could also be related to tumor cell metabolism. For example, leptin derived from mammary adipocytes was shown to induce fatty acid β-oxidation in BC stem cells, promoting resistance to the microtubule poison paclitaxel [9]. The role of ECM components has been highlighted *in vivo* by the group of Philipp Scherer [10]. They have shown that endotrophin, a cleavage product of collagen VI alpha 3 chain, enhanced epithelial–mesenchymal transition (EMT), causing resistance to cisplatin in a mouse mammary tumor model. Independently of their secretory function, adipocytes were described to absorb and efficiently metabolize daunorubicin in its inactive metabolite daunorubicinol, reducing its antileukemia effect in the local microenvironment [11]. This aspect needs to be investigated in BC. While these findings support that MAT can directly affect drug response in BC, a complete understanding of this effect is still lacking.

We recently investigated the mechanism of DOX resistance induced by mammary adipocytes and its regulation by obesity. Indeed DOX, a member of anthracycline family, is considered to be one of the most effective agents for BC treatment [12]. We found that cocultivating BC cells with adipocytes contributes to DOX resistance in a panel of human and murine cell lines, independently of their subtype [13]. Interestingly, cocultivated BC cells exhibit a multi-drug resistance (MDR) phenotype with resistance to paclitaxel and 5-fluorouracil. Although adipocytes contribute to increase DOX efflux in cocultivated cells, this was unrelated to the major ABC transporters function, a common mechanism of DOX resistance. We further demonstrated that this effect was linked to the increased expression of the transport-associated major vault protein (MVP). MVP contributed to DOX efflux by decreasing nuclear DOX associated with its accumulation in cytoplasmic vesicles, which are then expelled into the extracellular medium. Using a 3D culture system of isolated human mammary adipocytes from either lean or obese patients, we showed that all the depicted process was amplified by obesity. To our knowledge, this is one of the first studies exemplifying the direct impact of obesity on chemotherapy resistance in humans. Indeed, by means of a mouse model, obesity has been demonstrated to promote resistance of postmenopausal BC to the nucleoside analog gemcitabin that could be overcome by a nanoparticle formulation of gemcitabin [14]. One interesting finding in our study was that MVP expression was higher at the invasive front of human tumors, where cancer cells are at close proximity with adipocytes, than in the tumor center. Preliminary evidences suggest that this process could be amplified in obesity since we found a small, but not significant, enhancement of this effect in tumors from overweight/obese patients. It is noteworthy that numerous studies using TMA (tissue micro-arrays) mainly take into account the tumor center, therefore excluding the invasive front. This could lead to a loss of information especially when investigating the impact of obesity, since we consistently show that the cancer cells/adipocytes cross-talk occurs at the tumor invasive front including MVP overexpression, a drug resistance protein as stated above. Implication of MVP in the clinical response to chemotherapy needs to be confirmed using a dedicated collection including the tumor border in the studied samples. If these results were to be confirmed, they would clearly reinforce the interest in developing MVP inhibitors that are lacking at the moment.

Adipocytes probably also control the response to other BC therapies, such as targeted therapy and radiotherapy, although clinical evidence (and the impact of obesity) are limited. Adipocytes were shown to protect HER2-positive BC cells from trastuzumab-mediated cytotoxicity *in vitro* and from the antitumor effect of this antibody in mouse xenograft models [15]. By means of a diet induced murine model, obesity and excess energy were described to activate FGFR1, a known mediator of endocrine therapy resistance, in breast tumors [16]. Obesity was also shown to promote BC resistance to anti-VEGF therapy in mice models via the production of IL-6 and FGF-2, a pro-angiogenic factor [17]. Finally we have also demonstrated that adipocytes promote a radioresistant phenotype in BC cells by upregulating IL6 secretion by tumor cells [18].

Although some epidemiological studies and several biological mechanisms link obesity to BC prognosis, the impact of treatment resistance needs to be further investigated. Better knowledge of the impact of AT in this context will allow to identify new druggable targets and/or to adapt current therapeutic strategy in obese patients with BC.
